# Red urine, updated for the nephrologist: a case report

**DOI:** 10.1186/s12882-018-0939-9

**Published:** 2018-06-08

**Authors:** Alexandre Le Joncour, Laurent Mesnard, Alexandre Hertig, Thomas Robert

**Affiliations:** 1 Department of Nephrology and Renal Transplantation, Hôpital universitaire Tenon, AP-HP, Université Pierre et Marie Curie, Paris, France; 2 Department of Nephrology and Renal Transplantation, Hôpital universitaire de la Conception, AP-HM, Marseille, France; 3 Centre de néphrologie et transplantation rénale, APHM, Hôpital universitaire de la Conception, 147 Bd Baille, 13385 Marseille, France

**Keywords:** Sickle cell trait, Hematuria, Kidney concentrating ability, Proteinuria, Kidney papillary necrosis

## Abstract

**Background:**

Sickle cell trait is not completely benign, and some renal complications can occur. The baseline rate of admission for gross hematuria in normal males carrying the sickle cell trait is 2%.

**Case presentation:**

A 35-year-old non-smoking African man experienced a 2-week history of painless, profuse and persistent gross hematuria. Laboratory tests showed normal renal function, hematuria and mild proteinuria. Abdominal ultrasonography and computed tomography angiography revealed no renal abnormalities; the bladder appeared pristine under cystoscopy. The diagnosis of sickle cell trait associated with gross hematuria was made using hemoglobin electrophoresis; renal biopsy and its complications were avoided. Urine was clear after 2 weeks of oral hydration and gamma epsilon-aminocaproic acid.

**Conclusion:**

Hemoglobin electrophoresis should be performed in cases of gross hematuria. Coupled with other non-invasive evaluation, this could avoid renal biopsy and its associated complications.

## Background

Gross hematuria in young adults requires a complete work-up, including renal imaging with ultrasound, computed tomography angiography, cystoscopy and microbiological tests to rule out renal obstruction, kidney stones, renal infarction, schistosomiasis, other infections, toxic/herbal consumption and urological tumors. Renal biopsy is then often needed to explore glomerulopathies such as IgA nephropathy.

The prevalence of the sickle cell trait in the African black population is between 20 and 40% [1]. Although it is often asymptomatic, it can be responsible for specific renal abnormalities. Half of the cases of hematuria are due to renal papillary necrosis [2], although renal obstruction, urinary infection, kidney stones, renal infarction, papillary necrosis, schistosomiasis and urological tumors such as renal medullary carcinoma must be ruled out [3].

Only a few cases [4, 5] of sickle cell trait and gross hematuria have been described. We describe here a case of gross hematuria in a patient with unknown sickle cell trait that we feel should be brought to the attention of nephrologists.

## Case presentation

A 35-year-old non-smoking African man was admitted for a renal biopsy.

His medical history was of recurrent multiple sclerosis, identified 3 years previously and limited to loss of sensation of the extremities. Since diagnosis, he had been treated with a sphingosine 1-phosphate modulator. He was receiving no other treatment.

Two weeks after a common dental procedure, he experienced a sudden onset of painless, profuse and persistent gross hematuria. He did not take any medication such as a non-steroid anti-inflammatory drug or antibiotics. There were no other urinary symptoms, sore throat, skin rash, abdominal pain or bone pain. Physical examination revealed no abnormalities except gross hematuria without clots. Laboratory tests are shown in Table 1. Abdominal ultrasonography and excretory-phase computed tomography angiography revealed no renal abnormalities; the bladder appeared pristine under cystoscopy. Given these findings, origin of the hematuria was obscure. Significant proteinuria (around one gram per day) was found, and the patient was referred to a nephrologist for a renal biopsy in order to rule out glomerulopathy. However, the biopsy was not performed since a simple blood test established the diagnosis.

**Table 1:** Laboratory tests

Blood sample		Urinary sample	
Hb (g/L)	13.1	WBC	< 1000/ml
WBC (g/L)	3.2	Red blood cells	> 1,000,000/ml
Platelets (g/L)	232	Burr cells	90%
Reticulocytes (g/L)	42	Acanthocytes	Absent
Na + (mmol/L)	135	Bacterial culture	Negative
K+ (mmol/L)	4.2	Fungi culture	Negative
Cl- (mmol/L)	106	Schistosoma microscopy	Negative
HCO3- (mmol/L)	26		
Creatinine (μmol/L)	80	Na + (mmol/24 h)	184
Urea	4.8	K+ (mmol/24 h)	186
Haptoglobin (g/L)	1.21	Urea (mmol/24 h)	100
Lactate dehydrogenase (U/L)	177	Creatinine (mmol/24 h)	6.0
Schistosoma IgG	Negative	Proteinuria (g/24 h)	0.8

*HB* hemoglobin, *IgG* immunoglobulin G, *WBC* white blood cell

Indeed, hemoglobin electrophoresis revealed a sickle cell trait (HbAS) with 62% HbA and 34% HbS, respectively. The diagnosis of renal papillary necrosis or microscopic renal papillary necrosis with macroscopic hematuria related to sickle cell trait was made.

The patient was treated with oral alcalin hydration and gamma epsilon-aminocaproic acid. Urine was clear of hematuria after 2 weeks of treatment and there was no recurrence after 1 year.

## Discussion

Sickle cell trait is not completely benign, and can be responsible for specific renal abnormalities: micro- or gross hematuria, weak bladder, papillary necrosis, renal infarction, renal medullary carcinoma [6], increased risk of exertional rhabdomyolysis, chronic kidney disease and albuminuria [7]. Hematuria is the most common complication in male patients with the sickle cell trait compared to those with normal hemoglobin [2, 8]. Hematuria may originate from either kidney, although a preponderance of left-sided renal bleeding has been observed. Papillary necrosis is most common in subjects aged 30 to 40 years. Most frequently, the bleeding remits spontaneously with symptomatic treatment but may recur in 50% of cases. Rarely, hematuria requires multiple transfusions and is life-threatening. Recurrent papillary necrosis may contribute to the development of chronic kidney disease [9].

Sickle cell trait is caused by a single mutation involving the substitution of a single amino acid (Glu → Val) at the sixth position of the β-chain of a normal hemoglobin (HbA) molecule. This single-point mutation leads to the polymerization of the mutant hemoglobin (sickle hemoglobin or HbS) molecule and morphological deformation of red blood cells (RBCs) into rigid sickle shapes with abnormal rheology under deoxygenated conditions. Two major factors may promote HbS polymerization in the arterial blood passing through the long vasa recta of the renal medulla: First, the low partial pressure of oxygen leading to RBC sickling in the vasa recta; second, shrinkage of RBCs due to hypertonic medulla shifts water from the interior of the RBC, thus leading to HbS polymerization which is extremely sensitive to hemoglobin concentration. These factors which promote sickling by prolonging red cell microvascular transit times play a critical role in the initiation of a renal vaso-occlusive event. They result in microthrombi formation and infarction, which leads to microscopic papillary necrosis or frank papillary necrosis. Therefore, hematuria related to the sickle cell trait is urological and not glomerular in essence. Repeated microinfarctions in the renal medulla and changes in the renal blood flow disrupt the counter-current mechanisms leading to the relative loss of the concentrating ability of the kidney, namely impaired urinary concentration, which is dose dependent according to the percentage of sickle hemoglobin [10]. Of note, papillary necrosis is not always present on standard imaging [3].

Renal papillary necrosis presenting as isolated painless gross hematuria related to the sickle cell trait usually appears in patients aged between 20 and 50 years. Renal papillary necrosis is difficult to diagnose. Presence of sloughed papillae in the urine is pathognomonic but rarely seen. Sloughing papillae can also occasionally produce an obstruction to urine outflow and renal failure. A complete work-up, including renal imaging with ultrasound, computed tomography angiography and urography, cystoscopy and microbiological tests should be performed to rule out renal obstruction, kidney stones, renal infarction, schistosomiasis, other infections, toxic/herbal consumption and urological tumors such as rare but fatal renal medullary carcinoma.

Renal papillary necrosis is commonly managed conservatively with bed rest, intravenous hydration, urine alkalinization, and oral urea [7]. For severe bleeding, gamma epsilon-aminocaproic acid, desmopressin acetate infusion, direct ureteroscopic tamponade or fulguration may be occasionally required [11, 12]. Gamma epsilon-aminocaproic acid must be used with caution as it can predispose to clot formation and obstruction of the urinary system.

## Conclusion

Sickle cell trait, which confers malarial resistance by an unknown mechanism, has long been considered benign and asymptomatic. Hematuria is among the most common renal manifestations in both sickle cell trait and disease. Up to 50% of patients with sickle cell trait may develop papillary necrosis [2]. Sickle cell trait and renal involvement such as papillary necrosis as gross hematuria is not rare. In our case, isolated macroscopic hematuria was the initial presentation of sickle cell trait, which led to its diagnosis. Our case is an important reminder to nephrologists to avoid a renal biopsy and its adverse effects in black patients who have no apparent previous medical history [10].

## Abbreviation

RBC: Red blood cell
